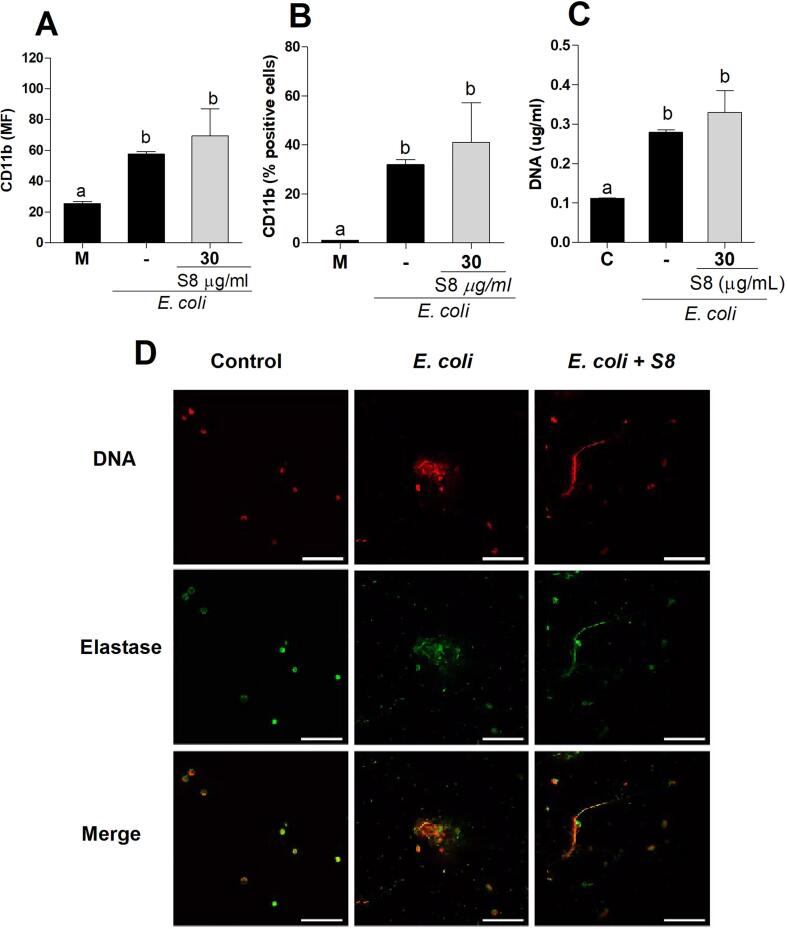# The phytoactive constituents of *Eugenia selloi* B.D. Jacks (pitangatuba): Toxicity and elucidation of their anti-inflammatory mechanism(s) of action

**DOI:** 10.1016/j.fochms.2022.100093

**Published:** 2022-03-07

**Authors:** Josy Goldoni Lazarini, Adna Prado Massarioli, Jackeline Cintra Soares, Bruno Dias Nani, Nancy Charo, Douglas Souza Oliveira, Lauren Camargo, Miryam Paola Alvarez-Flores, Isabel de Fátima Correia Batista, Ana Marisa Chudzinski-Tavassi, Severino Matias de Alencar, Marcelo Franchin, Pedro Luiz Rosalen

**Affiliations:** a Department of Biosciences, Piracicaba Dental School, University of Campinas, Limeira Avenue, 901, Areião, 13414-903 Piracicaba, SP, Brazil; b Department of Agri-Food Industry, Food and Nutrition, “Luiz de Queiroz” College of Agriculture, University of São Paulo, Pádua Dias Avenue, P.O. Box. 9, 13418-900 Piracicaba, SP, Brazil; c Laboratory of Experimental Thrombosis, Institute of Experimental Medicine, CONICET, National Academy of Medicine, 1425 Buenos Aires, Argentina; d Centre of Excellence in New Target Discovery (CENTD), Development and Innovation Laboratory, Butantan Institute, São Paulo, SP, Brazil; e Faculty of Dentistry, Federal University of Alfenas, Alfenas, MG, Brazil; f Biological Sciences Graduate Program, Federal University of Alfenas, Alfenas, MG, Brazil

**Keywords:** *Eugenia selloi*, Anti-inflammatory, ROS and RNS, Native fruit

## Abstract

We determined the phytochemical composition, anti-inflammatory mechanism of action, ROS/RNS scavenging capacity and systemic toxicity of a purified subfraction (S8) of *Eugenia selloi*. The composition of S8 was assessed by LC-ESI-QTOF-MS; the anti-inflammatory activity in RAW264.7 macrophages through NF-κB activation and biomarkers by multiplex in THP-1 cells; neutrophil migration, intravital microscopy and ICAM-1 expression in mice; NETs formation and CD11b expression; S8 scavenging capacity of ROS/RNS; toxicity in *Galleria mellonella* larvae model. Coumaric acid, quercetrin and vanillic acid were identified. S8 decreased NF-κB activation, IL-1β, IL-6, IL-10, MDC and MCP-1 levels, reduced neutrophil migration and ICAM-1 expression in mice; S8 did not interfere NET formation and CD11b expression, exhibited high antioxidant and showed negligible toxicity. *E. selloi* proved to be a promising, yet underexplored source of bioactive compounds, which can be useful employed in agribusiness and in the pharmaceutical and food industry to develop new products or human health supplies.

## Highlights

• Purified subfraction from *Eugenia selloi* fruit, showed anti-inflammatory activity.
• It were identified isomers of quercetrin, vanillic acid, and coumaric acid.
• S8 reduced NF-κB, IL-1β, IL-6, IL-10, MDC and MCP-1 levels in macrophages.
• S8 reduced neutrophil migration and ICAM-1 expression in mice.
• S8 showed scavenging capacity against ROO^•^, HOCl and NO^•^ biological radicals.

## 1 Introduction

Natural products are one of the most important sources of compounds with biological properties. The Brazilian Atlantic rainforest shelters 1–8% of the world’s biodiversity, accounting for as many as 20,000 species. Unfortunately, this ecosystem has been endangered over the last decades and is now under threat of extinction (Ribeiro et al., 2009).

*Eugenia* species (Myrtacea family) occur in the Atlantic rainforest and have a great potential for fresh fruit consumption or as an agro-industrial (juice, jam and ice cream), cosmetic, nutritional and pharmaceutical product, with outstanding biological properties (de Araújo et al., 2019, Lazarini et al., 2018, Soares et al., 2019). Some plants of the *Eugenia* genera were shown to have anti-inflammatory, antimicrobial, antioxidant, and anticancer activity (de Araújo et al., 2019, Infante et al., 2016, Sardi et al., 2017). A study conducted by Infante et al., 2016 revealed that the oral administration of pulp extracts of *E. brasiliensis* and *E. myrcianthes* reduced the neutrophil migration into the peritoneal cavity of mice, exerting an anti-inflammatory activity. The authors identified compounds such as quercetin, gallic acid, coumaric acid, and catechin/epicatechin that may be related to their anti-inflammatory activity.

The inflammatory process is orchestrated by several cell types. Tissue resident macrophages are activated by lipopolysaccharides (LPS)*,* thereby activating an important nuclear transcription factor for the immune response - Kappa B nuclear factor (NF-κB). In consequence of this activation, proinflammatory cytokines and chemokines are released, and rolling proteins (selectins, *e.g*., P and E) and adhesion proteins (e.g.*,* ICAM-1) are expressed to attract neutrophils, lymphocytes, and monocytes into the inflamed tissue (Maleki et al., 2019, Taniguchi and Karin, 2018).

Upon internalization of pathogens, defense cells produce reactive nitrogen species (RNS), reactive oxygen species (ROS), and different proteases, which are mainly involved in pathogen killing. However, if this process is exacerbated, it may lead to tissue damage and fibrosis and result in the development of chronic inflammatory diseases, such as rheumatoid arthritis, multiple sclerosis, psoriasis, asthma, cancer, atherosclerosis, and diabetes (Ashley et al., 2012, Peres et al., 2016).

Thus, the search for novel bioactive molecules that can control ROS/RNS production while modulating the inflammatory process through several pathways and molecular targets is much needed (Lazarini et al., 2016, Maleki et al., 2019).

*Eugenia selloi* B.D. Jacks (synonym *Eugenia neonitida* Sobral) is a Brazilian native fruit commonly known as “pitangatuba”. It measures up to 2.5 m and its fruit has an oblong shape, bright yellow color, and an intense bittersweet scent (de Araújo et al., 2019, Vilar et al., 2006). Most studies on *E. selloi* published to date have only characterized its physicochemical composition but not its biological potential. We hypothesized that the chemical compounds present in a purified subfraction of *E. selloi* can scavenge ROS/RNS and, consequently, modulate the inflammatory process. Thus, this study determined the phytochemical composition, anti-inflammatory mechanism of action, ROS/RNS scavenging capacity, and systemic toxicity of the purified subfraction of *E. selloi.*

## 2 Material and methods

### 2.1 Reagents

The following reagents were used in this study: formic acid (Tedia, Fairfield, OH, USA); purified water (Millipore Milli-Q System SAS, Molsheim, France); acetonitrile, methanol, and ethanol (J.T. Baker, Phillipsburg, USA); Roswell Park Memorial Institute (RPMI), lipopolysaccharide (LPS) from *Escherichia coli* 0111:B4, phorbol 12-myristate 13-acetate (PMA), DMSO (dimethylsulfoxide), carrageenan, dexamethasone, diaminofluorescein-2 (DAF-2), 3-(4,5-dimethylthiazol-2-yl)-2,5-diphenyltetrazolium bromide (MTT), (±)-6-hydroxy-2,5,7,8-tetramethylchroman-2-carboxylic acid (Trolox), sodium nitroprusside, sodium hypochlorite solution (NaOCl), nitrotetrazolium blue chloride (NBT), dibasic potassium phosphate, 2,2-azobis(2-methylpropionamidine) β-nicotinamide adenine dinucleotide (NADH), phenazine methosulfate (PMS), rhodamine 123, dihydrochloride (AAPH) and fluorescein sodium salt (Sigma-Aldrich, St. Louis, USA); fetal bovine serum (FBS) and penicillin/streptomycin (Gibco, Grand Island, USA); RAW 264.7 macrophages transfected with the NF-kB-pLUC gene (Applied Biological Materials Inc., Richmond, Canada); Silicagel 60 (0,063–0,200 mm) (MERCK KGaA, Frankfurt, Germany); luciferin (Promega Corporation, Madison, USA); Lysis buffer TNT, mixture of TRIS and Tween 20 (Amresco, Inc., West Chester, EUA). TNF-α and CXCL2/MIP-2 kits (R&D Systems, Inc, Minneapolis USA); LC-18 SPE cartridges 2 g (Supelco, Bellefonte, USA); antibodies ICAM-1 and α-tubulin (Santa Cruz Biotechnology, Texas, EUA); ficoll Hypaque (1.078 g/m density) (GE Healthcare, Buckinghamshire, UK); antibodies Rabbit anti-neutrophil elastase (Calbiochem-Merk Millipore, Darmstad, Germany); anti-rabbit Alexa 488 (Invitrogen Molecular Probes Eugene, EUA); secondary anti-mouse or goat peroxidase labeled IgG (Vector laboratories Inc., Burlingame, EUA); MILLIPLEX MAP Human Cytokine/Chemokine Magnetic Bead Panel (Merck KGaA, Darmstadt, Germany). Human monocytic cell line (THP-1) (American Type Culture Collection – ATCC, Manassas, USA); BV421-anti-CD11b antibody 1:160 (BioLegend, San Diego, USA); Quant-iT™ PicoGreen™ dsDNA Assay Kit (Invitrogen, Carlsbad, USA); column reversed phase chromatography (4.6 × 250 mm × 5 µm; Phenomenex Luna C18 column, California, USA).

### 2.2 Plant material and phytochemical extraction

Due to the *E. selloi* be a Brazilian native fruit, the Council for Genetic Heritage Management allowed the sample collection under permission CGEN #AD4B64F (Brazilian Ministry of Environment). The plants and fruits were collected several times between November and February 2016 in the Atlantic rainforest region by the municipality of ‘Campina do Monte Alegre’ (S 23°53′57″; W 48°51′24″), São Paulo State, Southeastern Brazil. The specimens were deposited under voucher number HPL 5279 in the herbarium of the “Luiz de Queiroz” College of Agriculture at the University of São Paulo (ESALQ/USP), Piracicaba, São Paulo. Firstly, the pulps *in natura* were washed, frozen, lyophilized (Cheimika vacuum Freeze Dryer, Salerno, Italy) and 50 g of *E. selloi* was extracted by ultra-sound plus a mixture of ethanol and water (80:20, v/v; respectively) by 30 min, evaporated and lyophilized until use. *E. selloi* extract was further fractionated by open dry column chromatography on normal phase silica gel using a mixture of ethyl acetate:methanol:water (77:13:10, v/v) to recover the fractions with different polarities. The most bioactive anti-inflammatory fraction was selected to a chemical refinement (subfractionation).

### 2.3 Phytochemical purification

Briefly, 100 mg of bioactive fraction were applied into a reverse phase C18 column. The elution initiated with a mixture of water:methanol (50:50, v/v) in a linear gradient between 10% and 100% of methanol. Twelve subfractions were obtained and named S1 to S12, which were monitored during the elution process under UV light (366 nm). In addition, all subfractions were examined by thin layer chromatography (TLC) under UV light at 366 nm wavelength. Ten subfractions were submitted to anti-inflammatory bioguided assays, *i.e.*, NF-κB activation, to select for the most bioactive subfraction, which was the S8. Dimethyl sulfoxide (DMSO) at 0.1% (v/v) was used as a solvent to dissolve S8 in all *in vivo* and *in vitro* assays. This subfraction was submitted to liquid chromatography coupled to high-resolution mass spectrometry analysis (LC-ESI-QTOF-MS, Bruker Daltonics, Massachusetts, EUA).

### 2.4 Phytochemical composition analysis

#### 2.4.1 High-Resolution mass spectrometry analysis (LC-ESI-QTOF-MS)

Twenty microliters of S8 were injected into the High-resolution mass spectrometry MAXIS 3G (Bruker Daltonics, Massachusetts, EUA) with a Z-electrospray (ESI) operating in negative ion mode with a nominal resolution of 60,000 *m*/*z*. An external calibration was carried out using the software MAXIS 3G – Bruker Daltonics 4.3 to check for mass precision and data analysis. The liquid chromatography (Shimadzu Co., Quioto, Tokyo) with a column reversed phase chromatography C18 column was used coupled with a quaternary pump (LC-20AD), photodiode array detector (SPD-20A), nebulizer at 2 Bar; temperature at 200 °C; dry gas at 8 L/min and HV at 4500 V. The running follow the conditions: mobile phase was perform with (A) water/formic acid (99.75/0.25, v/v) and (B) acetonitrile/formic acid/water (80/0.25/19.75, v/v/v). The flow rate was 1 mL/min, and the gradient was changed as follow: 10 % solvent B (0 min), to 30 % B (20 min), 50 % B (32 min), 95 % B (38 min), 95 % B (60 min), and reducing to 10 % B (75 min). The compounds identification was performed by comparison of mass spectra (MS/MS), molecular formula and exact mass available in the bank data and scientific literature (Soares et al., 2019).

### 2.5 *In vitro* anti-inflammatory assays

#### 2.5.1 Cell culture and MTT assay (murine lineage)

RAW 264.7 macrophages cells were cultured in RPMI 1640 medium supplemented with penicillin (100 U/mL), fetal bovine serum (FBS; 10 % v/v) streptomycin sulfate (100 μg/mL) and L-glutamine under 37 °C, 5 % CO_2_ conditions (Sanyo MCO-18AIC(UV) CO_2_ Incubator, Osaka, Japan). Macrophages were cultured at 5 × 10^5^ cells/mL in 96-well plate for 24 h. The cells were treated with S8 at 3, 10, 30, 100 and 300 µg /mL or medium (negative control) and incubated for an additional 24 h. All groups received the stimuli lipopolysaccharide (LPS) at 10 ng/mL, except for the negative control for 4 h. After, the supernatant was removed, and then added the MTT solution (0.3 mg/mL) in all wells. The plate was incubated for 3 h (37 °C, 5% CO_2_). The supernatant was discarded, 100 µL of DMSO were added each wells, and the absorbance measured at 470 nm using a microplate reader (SpectraMax M3, Molecular Devices, California, EUA) (Lazarini et al., 2018).

#### 2.5.2 Nuclear factor-κB activation and cytokines levels

Transfected RAW 264.7 macrophage were cultured at 3 × 10^5^ cells/mL with S8 at 10, 30 and 100 µg/mL. After 30 min, all experimental groups received LPS stimulation (10 ng/mL) for 4 h, except for the culture media control (negative control). Then, the macrophages were lysed using lysis buffer and luciferase reagent (luciferin at 0.5 mg/mL) were added. The luminescence was measured with a microplate reader (SpectraMax M3, Molecular Devices, California, EUA). Next, TNF-α, and CXCL2/MIP-2 levels were quantified, according to the manufacturers’ instructions. The results were expressed as pg/mL (Lazarini et al., 2018).

### 2.6 Systemic toxicity

#### 2.6.1 In vivo toxicity model

In order to verify the toxic effects of S8, we next determined the systemic acute toxicity in *G. mellonella* larvae model. *G. mellonella* larvae (200 to 300 mg) with no signs of melanization were randomly selected for each group (n = 15). A 10 µL aliquot of S8 (0.1; 0.3; 1; 3 and 10 mg/kg) or control (0.9% NaCl, w/v) was injected into the hemocoel of each larva via the last left proleg using a Hamilton syringe (Hamilton, Reno, EUA). The larvae were incubated at 30° C (Bio-Oxygen Demand incubator, SP labor, Sao Paulo, Brazil), and their survival was monitored at selected intervals for up to 72 h. Larvae with no movements upon touch were counted as dead (Soares et al., 2019).

### 2.7 *In vivo* anti-inflammatory assays

#### 2.7.1 Animals

specific-pathogen free (SPF) C57BL/6JUnib male mice, specific-pathogen free (SPF), four weeks old, weighing between 22 and 25 g, were purchased from the Multidisciplinary Center for Biological Research in Animal Science (CEMIB/UNICAMP) after approval by the Institutional Ethics Committee on Animal Research of the University of Campinas (CEUA/UNICAMP, Protocol Number 4371-1, approved on 09/23/2016). All animals were housed *in vivarium* under humidity (40–60%) and temperature (22 ± 2 °C) control in 12 h light–dark cycle, with access to food and water *ad libitum*. The animals were deprived of food for 8 h before oral administration of S8. This study was carried out in strict accordance with the guidelines for the care and use of animals.

#### 2.7.2 Neutrophil migration assay

Mice were administered orally (via gavage) single doses of S8 (3 and 10 mg/kg). The bioactive fraction which originated S8 (3 mg/kg) was used as an internal control. The animals in the positive control group received orally dexamethasone (2 mg/kg) and those in the negative control received 0.9% saline (vehicle). All animals, except those in the vehicle group, received carrageenan, i.p. (500 µg/cavity), 1 h after the oral treatment. After 4 h, the mice were sacrificed, and their peritoneal cavity was washed and recovered to count for the total number of leukocytes and neutrophils. The results were expressed as number of neutrophils *per* cavity.

#### 2.7.3 Cytokines levels

The cytokines levels of TNF-α and CXCL2/MIP-2 were determined by ELISA method according to the manufacturers’ instruction. The results were expressed in pg/mL.

#### 2.7.4 Intravital microscopy

Based on the results of the neutrophil migration assay, the S8 concentration of 3 mg/kg was selected for further anti-inflammatory testing by intravital microscopy (LEICA SP8 - mouse intravital microscopy, Wetzlar, Germany). Mice were pretreated orally with S8 at 3 mg/kg 60 min prior to an i.p. injection of carrageenan (500 µg/cavity). Leukocyte rolling and adhesion were rated by intravital microscopy after 2 h or 4 h of the inflammatory stimulus, as previously described (Baez, 1969, Fortes et al., 1992).

#### 2.7.5 Adhesion (ICAM-1) protein expression by western blotting

Mice were pretreated orally with S8 at 3 mg/kg in a single dose 1 h before i.p. administration of carrageenan (500 µg/cavity). After 4 h, the animals were sacrificed and their mesenteric tissues were exposed, isolated, and quantified by the Bradford method. Fifty micrograms of protein were transferred to a nitrocellulose membrane. Then, a solution containing 5% of nonfat milk was used to block the membrane for 1 h at 4 °C in TBS-T. After this period, the membrane was incubated with α-tubulin (1:500), as a loading control, and anti-ICAM-1 (1:500) overnight at 4 °C. The membrane was incubated with anti-mouse or anti-goat conjugated to peroxidase (1:5000) diluted in TBS-T containing 5% of nonfat milk for 1 h at room temperature. The bands of the specific antibody were visualized with chemiluminescence ECL for 60 s and exposed to a documentation system (UVITEC, Alliance Q9 Advanced – Imaging Systems, Cambridge, United Kingdom). Finally, a computer-based imaging system (ImageJ; National Institutes of Health, Bethesda, USA) was utilized to measure the intensity of the optical density of the bands.

### 2.8 Human cell culture assays

#### 2.8.1 Human macrophage cell culture (THP-1) and MTT assay

THP-1 cells was cultured in RPMI 1640 medium supplemented with 100U penicillin/streptomycin, 10% fetal bovine serum (FBS), 2 mM L-glutamine, 1 mM Sodium Pyruvate, and incubated at 37 °C, in 5% CO^2^ atmosphere. THP-1 cells were differentiated from monocytes to macrophages using phorbol 12-myristate 13-acetate (PMA). Briefly, cells were cultured in a 96-well plate (2 × 10^4^ cells/well) with PMA at 25 nM for 48 h, followed by 24 h of rest in a PMA-free medium. For the MTT assay, THP-1 were pretreated with S8 at 30, and 100 e 300 μg/mL for 1 h, followed by treatment with LPS at 10 ng/mL and incubation for 24 h. The control group received dexamethasone (positive control) at 1 μM, followed by treatment with LPS for 24 h. Next, the supernatant was removed and MTT solution (0.3 mg/mL) was added to the wells. The plates were incubated for 3 h (37 °C, 5% CO_2_). The supernatant was removed and 100 µL of DMSO were added to the wells. The absorbance was read at 470 nm in a microplate reader (SpectraMax M3, Molecular Devices, California, EUA) (Yu et al., 2020).

##### 2.8.1.1 Biomarkers multiplex assay

Cytokines, chemokines, and factors present in the supernatant of THP-1 cells were measured by customized multiplex magnetic bead-based assay HCYTOMAG-60K (MILLIPLEX MAP, Frankfurt, Germany). The following markers were detected: IL-6, IL-1β, TNF-α, VEGF, IL-8, IL-10, MDC, MCP-1, MIP-1α and MIP-1β.

#### 2.8.2 Isolation of human neutrophils

Neutrophils, also known as polymorphonuclear (PMNs) leukocytes, were collected and isolated from the peripheral blood of healthy donors. This study was conducted according to the guidelines of the Declaration of Helsinki. All patients signed an informed consent form to authorize the sample collection and data analysis (authorization protocol no. T.I.N°13167/19/X, Laboratory of Experimental Thrombosis, Institute of Experimental Medicine, CONICET, granted by Dra. Mirta Schattner, Buenos Aires, Argentina). Peripheral blood was withdrawn from healthy donors, PMNs were isolated, centrifuged (Centrifuge 5910 Ri Universal, Hamburgo, Germany) on Ficoll-Hypaque gradient (1078 g/ml density), obtained by dextran sedimentation and suspended in RPMI 1640 medium supplemented with 2% fetal bovine serum (contained >99.5% neutrophils, as determined by May-Grunwald-Giemsa-stained cytopreps). All assays were performed immediately after neutrophil isolation to avoid spontaneous cell activation (Charo et al., 2019).

##### 2.8.2.1 Neutrophil extracellular traps (NETs) formation assay: Immunochemical staining and quantification of extracellular DNA

For quantification purposes, human neutrophils (4 × 10^5^) were isolated, as described in the section 2.8.2, seeded onto 24-well plates, and treated with S8 at 30 µg/mL for 30 min. Next, the neutrophils were stimulated with *Escherichia coli* (1 × 10^5^) at a MOI (multiplicity of Infection) of 0.25, and cultured at 37 °C and 5% CO_2_ for 180 min. The DNA content released to the supernatants was measured using Quant-iT™ PicoGreen™ dsDNA Assay Kit in a fluorometer (BioTek Instruments, VT, California, USA). To obtain NETs images, after *E. coli* stimulation, the cells (untreated or pretreated with S8) were fixed with PFA (4 %), the permeabilization was carried out with Triton x-100 (0.25%), blocked with FBS (5 %) for 30 min, and stained with a rabbit anti-elastase (1:3000) antibody for 30 min. Then, the cells were incubated with a secondary anti-rabbit Alexa IgG-488 (1:2000) and propidium iodide (IP) (20 μg/ml) and mounted on glass slides with PolyMount. The images were analyzed by confocal fluorescence microscopy (Olympus FV-1000 microscope, Tokyo, Japan) coupled with a Plapon 60x/NA1·42 objective lens (Lapponi et al., 2013).

##### 2.8.2.2 CD11b neutrophil expression

Neutrophils were isolated as described in the Section 2.8.2. The cells (4 × 10^5^) were seeded and pre-treated with S8 at 30 µg/mL for 30 min. After this time, the neutrophils were stimulated with *E. coli* (1 × 10^5^) at a MOI of 0.25 for 2 h and then fixed and stained with BV421-anti-CD11b antibody 1:160 (M1/70). CD11b neutrophil expression was analyzed via fluorescence-activated cell sorting (BD FACSCalibur, Becton, USA) (Charo et al., 2019).

### 2.9 Deactivation of reactive oxygen and nitrogen species (ROS and RNS)

#### 2.9.1 Peroxyl radical (ROO^•^)

The ROO^•^ scavenging capacity of S8 was determined as previously described (Melo et al., 2015). Briefly, 30 µL of S8, 60 µL of fluorescein and 110 µL of an AAPH solution were transferred to a microplate. The reaction was performed at 37 °C and absorbance was measured every minute for 2 h at 485 nm (excitation) and 528 nm (emission) in a microplate reader (Molecular Devices, LLC, Sunnyvale, USA). Trolox standard was used at concentrations ranging from 12.5 to 400 µM. The results were expressed as µmol/Trolox equivalents per g of extract/subfraction (S8).

#### 2.9.2 Superoxide anion (O_2_^•−^)

The capacity of S8 to scavenge O_2_^•−^ generated by the NADH/PMS system was also determined. Aliquots of 100 µL of NADH, 50 µL of NBT, 100 µL of S8 and 50 µL of PMS were added to the wells of a microplate. The assay was performed at 25 °C and absorbance was measured at 560 nm after 5 min. A control was prepared replacing the sample with the buffer, and a blank was prepared for each sample dilution replacing PMS and NADH with the buffer. Absorbance was measured in a microplate reader (Molecular Devices, LLC, Sunnyvale, USA) and the results were expressed as IC_50_, that is, the mean quantity (µg/mL) of S8 required to quench 50 % of the superoxide radicals (Melo et al., 2015).

#### 2.9.3 Hypochlorous acid (HOCl)

The HOCl scavenging capacity of S8 was measured based on HOCl-induced oxidation of dihydrorhodamine 123 (DHR) to rhodamine 123, with modifications. HOCl was prepared using a 1% NaOCl solution, adjusting the pH to 6.2 by adding 10% H_2_SO_4_ solution. This solution was prepared in 100 mM phosphate buffer (pH 7.4) and its concentration was measured at 235 nm using the molar absorption coefficient 100 M/cm. The reaction mixture contained the S8 (7.5 µg/mL), 100 mM phosphate buffer (pH 7.4), 1.25 µM DHR, and 5 µM HOCl, with a final volume of 300 µL. The assay was carried out at 37 °C and fluorescence measurements were taken in a microplate reader (Molecular Devices, LLC, Sunnyvale, USA) at 528 ± 20 nm (emission) and 485 ± 20 nm (excitation). The results were expressed as IC_50_ (µg/mL) of S8 (Melo et al., 2015).

#### 2.9.4 Nitric oxide (NO^•^)

The nitric oxide (NO^•^) activity of S8 was determined using diaminofluorescein-2 (DAF-2) as a NO^•^ probe. Briefly, 50 µL of S8, 50 µL of SNP solution, 50 µL of buffer and 50 µL of DAF solution were added to the wells of a 96-well microplate. Changes in fluorescence (excitation = 495 nm, emission = 515 nm) were measured in a microplate reader (Molecular Devices, LLC, Sunnyvale, CA, USA) over a 120-min period at 5-min intervals. The results were expressed as IC_50_ (µg/mL) of S8 (Soares et al., 2019).

### 2.10 Statistical analysis

The results were expressed as mean ± standard deviation (SD). The data were checked for normality and homogeneity of variance. The differences between groups were analyzed using one-way or two-way analysis of variance (ANOVA), followed by Tukey’s or Bonferroni’s post-hoc tests. Survival curves of treated and untreated larvae were compared using the Log-rank (Mantel-Cox) test. The results were considered significant at p < 0.05.

## 3 Results

### 3.1 Chemical composition analysis

*Total phenolic content and High-Resolution Mass Spectrometry analysis (LC-ESI-QTOF-MS).* The chemical composition of S8 was tentatively identified by exact masses, MS/MS spectra and molecular formula. As seen in Table 1, the chemical analysis revealed the presence of quercetin-3-*O*-rhamnoside (quercitrin), vanillic acid-*O*-hexoside, and coumaric acid-*O*-hexoside. Full scan MS^1^ and fragment-ion spectra (MS^2^) for each identified peak of these compounds can be found in supplementary Fig. 1.

**Table 1:** Chemical Identification of S8 by LC-ESI-QTOF-MS analysis.

Compound	Rt (min)	Molecular formula	[M–H]^−^	MS^2^ fragments *(m*/*z)*
Quercetin-3-*O*-rhamnoside (quercitrin) I	22.2	C21H20O11	447.0548	**299.9899**, 447.0536
Quercetin-3-*O*-rhamnoside (quercitrin) II	22.2	C21H20O11	895.1163 [2M–H]^−^	**447.0546**, 299.9894
Quercetin-3-*O*-rhamnoside (quercitrin) III	22.4	C21H20O11	447.0549	**299.9899**, 447.0548
Quercetin-3-*O*-rhamnoside (quercitrin) IV	22.4	C21H20O11	895.1164 [2M–H]^−^	**447.0504**, 299.9904, 300.9976
Quercetin-3-*O*-rhamnoside (quercitrin) V	22.9	C21H20O11	447.0546	**299.9897**, 447.0545
Quercetin-3-*O*-rhamnoside (quercitrin) VI	23.1	C21H20O11	447.0549	**299.9899**, 300.9964, 447.0553
Quercetin-3-*O*-rhamnoside (quercitrin) VII	23.5	C21H20O11	447.0548	**299.9892**, 447.0526
Quercetin-3-*O*-rhamnoside (quercitrin) VIII	23.6	C21H20O11	447.0544	**299.9900,** 447.0530
Vanillic acid-*O*-hexoside I	31.7	C14H18O9	329.2319	**329.2313,** 171.1010**,** 167.1294
Vanillic acid-*O*-hexoside II	32.1	C14H18O9	329.2316	**329.2313,** 171.1023, 167.1078
Vanillic acid-*O*-hexoside III	32.3	C14H18O9	329.2320	**329.2305, 171.1010,** 166.8398
Coumaric acid-*O*-hexoside I	45.7	C15H18O8	325.1835	**183.0118,** 163.6136, 145.2988**,** 119.0504
Coumaric acid-*O*-hexoside II	46.5	C15H18O8	325.1831	**183.0107,** 163.6418, 145.2988, 119.0518

Rt = retention time; bold values correspond to the main fragments; negative ionization mode.

**Fig. 1:**
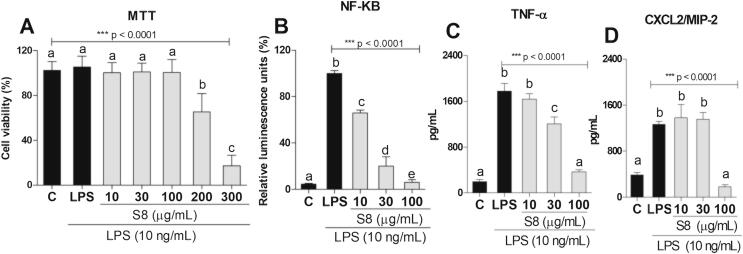
The effects of S8 on RAW 264.7 macrophage viability, and its anti-inflammatory activity. The groups were: Control (C, vehicle – DMSO 0.1%, (v/v) and S8 at several concentration (A) Percent viability of LPS-stimulated RAW 264.7 cells treated with S8 at 3, 10, 30, 100 and 300 μg/mL for 24 h. (B) NF-kB activation of LPS-stimulated macrophages treated with S8 at 10, 30, 100 μg/mL. (C and D) TNF-α, and CXCL2/MIP-2 levels in LPS-stimulated RAW 264.7 macrophages treated with S8 at 10, 30, 100 μg/mL. The results were expressed as mean ± SD, *n* = 4–6. Different letters indicate statistically significant differences intragroup, and the symbols *, ** or *** indicate statistical differences (p < 0.01; p < 0.001; and p < 0.0001, respectively) compared to the negative control group (one-way ANOVA followed by Tukey’s posthoc test).

### 3.2 Anti-inflammatory activity

S8 did not affect cell viability compared to the control up to 100 µg/mL (p > 0.05) in the control (C) RAW 264.7 macrophages, however, the concentrations of 200 and 300 µg/mL, induced significantly cell death when compared to the control (Fig. 1A).

As seen in Fig. 1B, cells treated with S8 at 10, 30 and 100 μg/mL had a significant decrease in NF-κB activation (34%, 76% and 93%, respectively) as compared to the LPS group (p < 0.0001). As seen in Fig. 1C, S8 significantly decreased TNF-α release at 30 and 100 μg/mL (32% and 80%, respectively) as compared to the LPS group and, CXCL2/MIP-2 release at 100 µg/mL (p > 0.0001).

### 3.3 *In vivo* analysis of systemic toxicity and inflammation

**Fig. 2:**
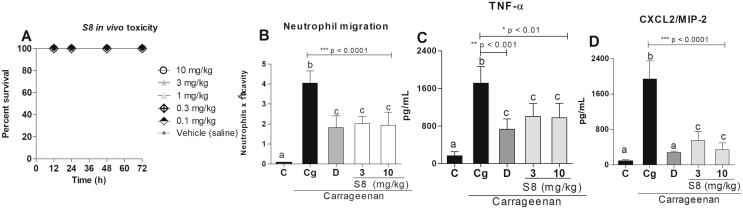
The effects of S8 on the systemic toxicity of *Galleria mellonella* larvae, and its anti-inflammatory activity *in vivo*. (A) Larvae were treated with S8 at 0.1, 0.3, 1, 3 and 10 mg/kg or control (vehicle DMSO 0.1%, v/v) and had their survival monitored over 72 h. (B) Effects of the treatments with the vehicle DMSO 0.1%, (v/v) (C), carrageenan (Cg), dexamethasone (2 mg/kg) and S8 at 3 and 10 mg/kg on neutrophil migration into the peritoneal cavity of mice induced by i.p. administration of carrageenan. (C and D) Effects of the treatments on the release of TNF-α and CXCL2/MIP-2 in mice. The results were expressed as mean ± SD, *n* = 4–6. Different letters indicate statistically significant differences intragroup, and the symbols *, ** or *** indicate statistical differences (p < 0.01; p < 0.001; and p < 0.0001, respectively) compared to the negative control group (one-way ANOVA followed by Tukey’s posthoc test).

As seen in Fig. 2A, systemic treatment with S8 did not have any toxic effects on *G. mellonella* larvae at doses up to 10 mg/kg. Different doses were chosen for this assay to determine which would be ideal for the *in vivo* assays in the mice model. As seen in Fig. 2B, animals pretreated orally with S8 at 3 and 10 mg/ kg showed a significant decrease in neutrophil migration (48% and 52%, respectively) when compared with those of the carrageenan control group (p < 0.0001).

In addition, treatment with dexamethasone (a gold standard control) led to a significant decrease (55%) in neutrophil migration. Interestingly, there was no statistical difference in neutrophil influx between mice treated with S8 (at both doses) and dexamethasone, a gold-standard corticosteroid widely used in medical and dental care (p > 0.05). This indicates that although S8 is not an isolated compound, but a mixture of compounds, its inhibitory activity on neutrophil influx reduced the inflammatory process and promoted health. In addition, mice treated with S8 at 3 and 10 mg/kg (Fig. 2C and D) showed a significant decrease in TNF-α (41% and 43%, respectively) and CXCL2/MIP-2 levels (60% and 80%, respectively) in their peritoneal cavity when compared with those of the carrageenan group. Animals treated with the control dexamethasone also showed decreased TNF-α (57%; p < 0.001) and CXCL2/MIP-2 levels (80%; p < 0.0001) when compared to their respective control.

### 3.4 Intravital microscopy and ICAM-1 expression

**Fig. 3:**
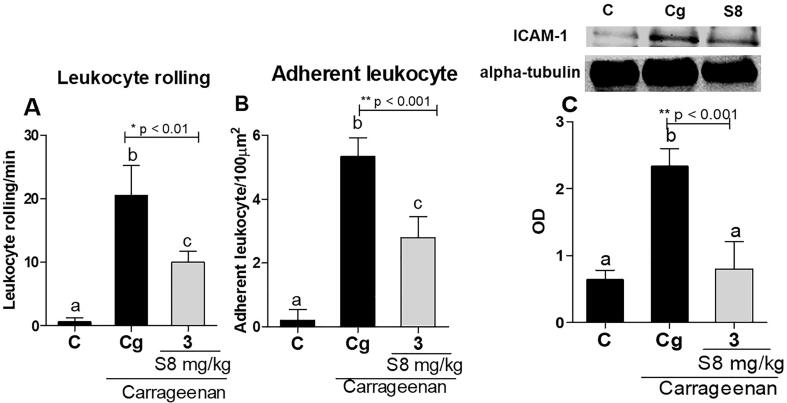
The inhibitory effects of S8 on leukocyte rolling and adhesion by intravital microscopy analysis and inhibition of ICAM-1 expression in mice. (A and B) Mice were pretreated with vehicle DMSO 0.1% (v/v) (C) or S8 at 3 mg/kg 60 min before carrageenan (Cg; 500 μg/cavity) injection. Cells were counted as the number of rolling and adherent leukocytes in the vessel. (C) ICAM-1 expression in endothelial cells of mice 3.5 h after i.p. injection of carrageenan. The optical densities of ICAM-1 bands were normalized to those of α-tubulin bands and are represented as arbitrary units of an optical density ratio. The results were expressed as mean ± SD, n = 6. Different letters indicate statistically significant differences intragroup, and the symbols *, ** or *** indicate statistical differences (p < 0.01; p < 0.001; and p < 0.0001, respectively) compared to the negative control group (one-way ANOVA followed by Tukey’s posthoc test).

In order to elucidate in real time the mechanism by which S8 decreases neutrophil influx into endothelial cells, we carried out an intravital microscopy assay and analyzed ICAM-1 expression in endothelial cells*.* Treatment with S8 at 3 mg/kg significantly decreased leukocyte rolling by 52% (Fig. 3A) and adhesion by 47% (Fig. 3B) in the mesenteric microcirculation of mice after carrageenan injection. Our findings further proved mechanistically that S8 reduces ICAM-1 expression (65%) in endothelial cells (Fig. 3C).

### 3.5 Anti-inflammatory activity in human macrophage-like cells

**Fig. 4:**
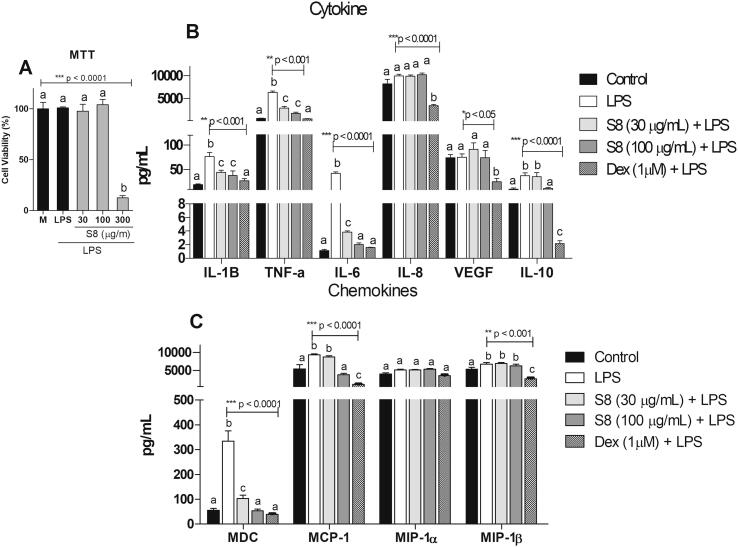
The effects of S8 on the viability of THP-1 cells and inflammation-related biomarkers*.* (A) Percent viability of THP-1 macrophages pretreated with control vehicle (DMSO 0.1%, (v/v), S8 at 30 and 100 μg/mL 1 h before LPS challenge for 24 h. (B) THP-1 macrophages pretreated for 24 h with culture media (control), LPS (10 ng/mL), S8 at 30 and 100 μg/mL, and dexamethasone (Dex) at 1 μM, 1 h before LPS challenge, except for the control group. The results were expressed as mean ± SD, *n* = 4. Different letters indicate statistically significant differences intragroup, and the symbols *, ** or *** indicate statistical differences (p < 0.01; p < 0.001; and p < 0.0001, respectively) compared to the negative control group (one-way ANOVA followed by Tukey’s posthoc test).

As seen in Fig. 4A, treatment with S8 at 30 and 100 μg/mL did not decrease the viability of THP-1 cells as compared to the control group (p > 0.05), however S8 affected cell viability at 300 μg/mL (p < 0.0001). In Fig. 4B shows that treatment with S8 at 30 µg/mL and 100 µg/mL reduced the levels of IL-1β, TNF-α, IL-6, and MDC as compared to their corresponding LPS controls (p < 0.001). At 100 µg/mL, S8 also reduced MCP-1 levels as compared to the LPS control (p < 0.0001). As expected, treatment with dexamethasone resulted in reduced levels of all tested cytokines and chemokines (p < 0.05), except for MIP-1α compared to its LPS control (p > 0.05).

### 3.6 CD11b expression and NET formation

As seen in supplementary Fig. 2A and B, neutrophils treated with S8 at 30 µg/ml did not show reduced CD11b expression as compared to the control induced by *E. coli* (p > 0.05). We demonstrated that S8 did not modulate CD11b expression induced by *E. coli* (supplementary Fig. 2A-B). However, it did decrease neutrophil migration into the peritoneal cavity of mice, suggesting that the anti-inflammatory activity of S8 may be associated to a specific cell type as it modulates adhesion molecules in endothelial cells. As for NET formation, treatment with S8 at 30 µg/mL did not reduce the release of *E. coli*-induced extracellular DNA as compared to the *E. coli-*induced control group (supplementary Fig. 2C; p > 0.05). To confirm the results obtained, we labeled NETs with PI to identify DNA and elastase. Then, NETs were visualized by confocal fluorescence microscopy. As expected, the supplementary Fig. 2D shows images corresponding to NETs formation induced by *E. coli*, which was not affected by S8 treatment.

### 3.7 Scavenging activity of S8 on peroxyl radical (ROO^•^), superoxide anion (O_2_^•−^), hypochlorous acid (HOCl) and nitric oxide (NO^•^)

The radicals HOCl and NO^•^ were expressed as IC_50_, that is, the amount of subfraction required to quench 50 % of ROS/RNS radicals. As seen in Table 2, S8 showed antioxidant activity on reactive radicals, as follows: 180 ± 0.01 μmol TE/g S8 (ROO^•^), 2.43 ± 0.15 μg/mL (HOCl) and 3.9 ± 0.15 μg/mL (NO^•^). However, it was no possible to determine the IC_50_ of S8 for the superoxide anion (O_2_^•−^).

**Table 2:** Antioxidant activity of S8 on peroxyl radical (ROO^•^), superoxide anion (O_2_^•−^), hypochlorous acid (HOCl) and nitric oxide (NO^•^).

Sample	ROO^•^ µmol TE/g extract	O_2_^•−^ µg/mL	HOCl µg/mL	NO^•^ µg/mL
S8	180 ± 0.01	nd	2.43 ± 0.15	3.9 ± 0.15

ROO^•^ is expressed as µmol TROLOX/g extract, nd = no activity was detected within the tested concentration range (14 pg/mL to 500 µg/mL); HOCL and NO^•^ were expressed as IC_50_ (µg/mL); mean ± SD; n = 3.

## 4 Discussion

In this bioguided study, we demonstrated that the *E. selloi* purified subfraction (S8) (i) has anti-inflammatory activity by decreasing neutrophil migration, NF-κB activation, cytokine and chemokine release, and ICAM-1 expression *in vivo;* (ii) has antioxidant activity by scavenging ROS/RNS; and (iii) contains polyphenolic compounds such as quercetin-3-*O*-rhamnoside (quercitrin), vanillic acid-*O*-hexoside, and coumaric acid-*O*-hexoside. HPLC-DAD-ESI-MS analysis revealed the presence of eight quercetin-3-*O*-rhamnoside isomers with precursor ion at *m*/*z* 447. The second-generation product ion spectra of the precursor ion at *m*/*z* 447.0549 [M–H]^−^ fragmented further to aglycone (*m*/*z* 300.9964/301) by losing a rhamnosyl unit (146 amu) (Hashim et al., 2012).

The tentatively identified hydroxybenzoic acids were three isomers of vanillic acid-*O*-hexoside (*m*/*z* 329), which showed typical fragmentation patterns by the loss of 162 Da (glycosidic moiety). This compound originated from deprotonated vanillic acid aglycones (*m*/*z* 167) and resulted in ion fragments at *m*/*z* 108 ([M–H–162–CH_3_–CO_2_]^−^) (Fischer et al., 2011).

As for hydroxycinnamic acids, two isomers of coumaric acid-*O*-hexoside were detected in S8 (RT = 45.7 and 46.5), which showed a loss of the sugar moiety [M–H–162]^−^ to yield coumaric acid (typical 163 → 119 fragmentation) (Vallverdú-Queralt et al., 2010).

The S8 subfraction is obtained through chemical fractionation of *Eugenia selloi* pulp extract. The chemical composition of the pulp extract and its fraction is published elsewhere (Lazarini et al., 2020). Bioactive fraction named F3 was submitted to subfractionation for chemical and biological refinement purposes. The subfractionation generated several samples and among them, S8 showed anti-inflammatory activity. The chemical refinement process of F3 generated S8, which became more concentrated and enriched containing eight quercitrin isomers, two coumaric acid isomers, and three vanillic acid isomers. Interestingly these last two compounds were not detected in F3 due to their low concentration but were detected in S8.

This is the first study determining the phenolic composition of an *E. selloi* subfraction (S8) by HPLC-MS/MS. After elucidating the chemical composition of the subfraction, we next tested the sample for its toxicity *in vitro* and anti-inflammatory activity. S8 showed strong anti-inflammatory activity in a macrophage cell model. Macrophages are innate immune cells that produce cytokines and chemokines that recruit inflammatory cells and initiate adaptive immune responses (Gong et al., 2019).

For macrophages to become active, stimuli such as LPS have to bind to the macrophage surface Toll-like receptor 4 (TLR-4), triggering intracellular signaling pathways that activate the nuclear transcription factor (*e.g*., NF-κB and others) (Gong et al., 2019). Once activated, NF-κB induces the expression of pro-inflammatory genes that consequently increase the release of cytokines and chemokines, matrix metalloproteinases (MMPs), and the expression of selectins and integrins, which are responsible for leukocyte rolling and adhesion towards the inflammatory focus (Taniguchi & Karin, 2018).

We next carried out *in vivo* assays to determine the systemic toxicity of S8 in *G. mellonella* larvae and its anti-inflammatory activity in mice. None of the doses tested in the larvae were toxic. This can be related to the presence of quercetin (without the rhamnoside portion) in the extract, which could exert some protective (antioxidant) effects in the larvae. *G. mellonella* is a widely accepted and validated model which can be used for toxicological screening of drugs. This approach has a low-cost, generates rapid results and, most importantly, reduces the number of animals for experimentation as its results correlate with those observed in mammals (vertebrates) in terms of ROS production, phagocytic hemocytes etc (Malmquist et al., 2019, Rochelle et al., 2016).

Given that S8 had non-toxic effects in the larvae, we next initiated the anti-inflammatory assays. Mice treated orally with S8 at 3 and 10 mg/kg had a decrease in the neutrophil migration into the inflammatory site (peritoneal cavity).

Neutrophils are the most abundant cells in the human innate immune system and can be recruited by diapedesis into the inflammatory focus via the expression of selectins and integrin ligands on the endothelium (Peres et al., 2016). Thus, the discovery of novel drugs or strategies able to control excess leukocyte influx into the inflammatory site would be a great contribution to the field of medicine.

Our data also showed that S8 inhibited NF-κB activation *in vitro,* and consequently, reduced TNF-α and CXCL2/MIP-2 levels*.* This was also observed in the *in vivo* inflammatory assays in mice. Authors found that rats chronically treated with quercitrin, a flavonoid also identified in S8, had reduced levels of TNF-α and showed a protective effect in induced colitis (Dönder et al., 2018). In another *in vivo* colitis model, the authors found that quercetrin decreased the recruitment of macrophages and neutrophils as well as iNOS and NF-κB expression (Camuesco et al., 2004). Lastly, another study reported that quercetrin reduced the progression of colitis in rats by decreasing MPO (myeloperoxidase) and iNOS levels (Comalada et al., 2005).

Vanilic acid can undergo extensive metabolization, and once its resulting metabolites circulate in the bloodstream, they may produce biological effects more potent than those of their precursors. In a previous study, the authors showed that a vanillic acid metabolite (vanillic acid glucuronide) significantly reduced the release of TNF-α in THP-1 human cells (di Gesso et al., 2015).

Thus, to prove in real time the mechanism by which S8 decreases the neutrophil influx into endothelial cells, we carried out an intravital microscopy assay and analyzed ICAM-1 expression in endothelial cells. Mice had decreased neutrophil adhesion, rolling and ICAM-1 expression after treatment with S8, indicating an anti-inflammatory activity in real time after 1.5 h of oral administration. The adhesion molecule ICAM-1 is expressed constitutively in low levels on the surface of vascular endothelial cells, lymphocytes and monocytes. Cytokines released by macrophages can stimulate endothelial cells to express ICAM-1 for leukocyte adhesion onto the vascular endothelium (Peres et al., 2016). Meng et al. (2016) reported that quercetin decreased the number of leukocytes recruited to lung tissues and inhibited ICAM-1 expression.

We further examined the effects of the S8 subfraction on a panel of inflammation-related biomarkers in human macrophage-like cells (differentiated from THP-1 cells).

Collectively, our data showed that S8 had an anti-inflammatory effect by reducing the release of LPS-induced pro-inflammatory markers such as IL-1β, TNF-α, IL-6, and MCP-1. LPS challenge is known to activate macrophages via the TLR4 receptor, leading to NF-kB pathway activation and, consequently, inducing a pro-inflammatory phenotype into M1 macrophage type (Wang et al., 2014). Salaverry et al. (2020) showed that *S. campestris* extract, which contained catechin and glycosylated derivatives of quercetin, reduced the levels of IL-1β, IL-6, and TNF-α, pro-inflammatory cytokines, after LPS-stimulated THP-1 human macrophages. Moreover, di Gesso et al. (2015) evaluated the anti-inflammatory activity of isolated flavonoids and found that vanillic acid reduced TNF-α release in THP-1 cells.

Taken altogether, S8 decreased the levels of cytokines and chemokines in macrophages (RAW 264.7), showed anti-inflammatory activity in mice (*in vivo*) and reduced inflammatory biomarkers in THP-1 cells, suggesting that S8 may also decrease biomarkers in human cells.

We hypothesized that S8 may diminish the neutrophil influx by interfering with membrane receptors on the surface neutrophils and not only with endothelial cells. This could affect intracellular signal transduction pathways and consequently influence CD11b expression and neutrophilic functions, such as NETs formation. NETs composition is based on chromatin associated with histones (majority), elastase, myeloperoxidase, and others, and is considered a protective mechanism against a broad range of microorganisms and have been related to an exacerbated inflammatory response and multiorgan failure (Colón et al., 2019, Lapponi et al., 2013). Hence, we determined the effects of S8 on CD11b expression on the surface of neutrophils and NET formation induced by *Escherichia coli.* Our results suggested that the anti-inflammatory activity observed was not directly related to NET formation nor CD11b expression.

Overall, our findings showed that S8 reduced NF-κB activation, leading to a decrease in TNF-α and CXCL2/MIP-2 levels, neutrophil rolling and adhesion, expression of ICAM-1 in the endothelium and, consequently, decrease in the neutrophil influx into the inflammatory site. The anti-inflammatory activity of S8 is likely to be related to the presence of isomers of quercetin, vanillic acid, and coumaric acid, which can act synergistically to modulate the inflammatory process, however, the hypothesis that some of them can be acting alone should not be disregarded.

As the anti-inflammatory activity of S8 can also be related to its antioxidant properties, we next carried out a series of assays to determine its ROS/RNS scavenging capacity*.*

There is evidence that flavonoids, such as quercetin isomers, have an anti-inflammatory effect due to their antioxidant properties. During the inflammatory process, neutrophils release ROS/RNS, such as superoxide radical O_2_^•−^, hydrogen peroxide (H_2_O_2_), hypochlorous (HOCl), nitric oxide (NO^•^) and others, which increase oxidative stress and activate the NF-κB pathway. Studies have shown that the excess of neutrophils contributes to tissue damage due to the presence of ROS/RNS, resulting in the onset of several conditions, such as diabetes, Alzheimer, cardiovascular diseases, cancer and others (Glennon-Alty et al., 2018, Luster et al., 2005, Muñoz and Costa, 2013). S8 showed strong antioxidant activity against ROO^•^, HOCL, and NO^•^. ROS/RNS scavenging is a defense mechanism consisting of several systems, such as superoxide dismutase, catalase, glutathione peroxidase, and glutathione reductase.

Thus, the S8 subfraction of *E. selloi* could be an interesting therapeutic alternative (as an antioxidant) to treat diseases associated with oxidative stress.

## 5 Conclusions

In summary, a nontoxic subfraction of *E. selloi* (S8) inhibited neutrophil migration by reducing NF-κB activation, which, as a result, reduced cytokine and chemokine release and ICAM-1 expression *in vivo*. Furthermore, S8 reduced the levels of inflammatory biomarkers in human macrophages and showed ROS/RNS scavenging capacity due to the presence of polyphenolic compounds. *E. selloi* can be classified as a superfruit as it meets antioxidant and anti-inflammatory requirements. *E. selloi* has gained attention as a promising source of bioactive compounds, and its consumption can be highly beneficial to human health (functional foods).

## 6 Funding sources

This study was supported by the São Paulo Research Foundation (FAPESP, grant numbers 2016/02926-6 and 2017/09898-0); the National Council for Scientific and Technological Development (CNPq, Grant # 310522/2015-3 and 307893/2016-2) and by the Coordenação de Aperfeiçoamento de Pessoal de Nível Superior - Brasil (CAPES) - Finance Code 001.

### CRediT authorship contribution statement

**Josy Goldoni Lazarini:** Methodology, Writing – original draft. **Adna Prado Massarioli:** Supervision. **Jackeline Cintra Soares:** Methodology. **Bruno Dias Nani:** Methodology. **Nancy Charo:** Methodology, Writing – review & editing. **Douglas Souza Oliveira:** Methodology, Writing – review & editing. **Lauren Camargo:** Methodology. **Miryam Paola Alvarez-Flores:** Methodology, Supervision, Writing – review & editing. **Isabel de Fátima Correia Batista:** Supervision, Writing – review & editing. **Ana Marisa Chudzinski-Tavassi:** Supervision, Writing – review & editing. **Severino Matias de Alencar:** Conceptualization, Funding acquisition, Supervision, Writing – review & editing. **Marcelo Franchin:** . **Pedro Luiz Rosalen:** Conceptualization, Funding acquisition, Supervision, Writing – review & editing.

## Declaration of Competing Interest

The authors declare that they have no known competing financial interests or personal relationships that could have appeared to influence the work reported in this paper.

## Figures and Tables

**Supplementary figure 1:**