# Supplementary material for: The phytoactive constituents of Eugenia selloi B.D. Jacks (pitangatuba): Toxicity and elucidation of their anti-inflammatory mechanism(s) of action
Source: Food Chem (Oxf). 2022 Mar 7;4:100093. doi: 10.1016/j.fochms.2022.100093 (PMC8991978; doi:10.1016/j.fochms.2022.100093)
Supplement: Supplementary data 1 [file mmc1.docx]

**1**

**2**

**3**

**4**

**5**

**6**

**7**

**8**

**9**

**10**

**11**

**A**

Supplementary figure 1: LC-MS/MS analysis of a purified subfraction (S8) of *Eugenia selloi*. Total ion count (TIC) of the chromatogram (A). Quercetin-3-*O*-rhamnoside I (1), Quercetin-3-*O*-rhamnoside II (1), Quercetin-3-*O*-rhamnoside III (2), Quercetin-3-*O*-rhamnoside IV (2); Quercetin-3-*O*-rhamnoside V (3), Quercetin-3-*O*-rhamnoside VI (4), Quercetin-3-*O*-rhamnoside VII (5), Quercetin-3-*O*-rhamnoside VIII (6), Vanillic acid-*O*-hexoside I (7), Vanillic acid-*O*-hexoside II (8), Vanillic acid-*O*-hexoside II (9), Coumaric acid-*O*-hexoside I (10), and Coumaric acid-O-hexoside II (11).

**1**

**B**

**C**

**C**

Full scan MS^1^ spectra at 22.2 min (B), and fragment-ion spectra (MS^2^) for the two major peaks (C). Number 1 refers to supplementary figure 1.

**2**

**B**

**C**

**C**

Full scan MS^1^ spectra at 22.4 min (B), and fragment-ion spectra (MS^2^) for the two major peaks (C). Number 2 refers to supplementary figure 1.

**3**

**B**

**C**

**C**

Full scan MS^1^ spectra at 22.9 min (B), and fragment-ion spectra (MS^2^) for the two major peaks (C). Number 3 refers to supplementary figure 1.

**4**

**B**

**C**

**C**

Full scan MS^1^ spectra at 23.1 min (B), and fragment-ion spectra (MS^2^) for the two major peaks (C). Number 4 refers to supplementary figure 1.

**5**

**B**

**C**

**C**

**C**

**C**

Full scan MS^1^ spectra at 23.5 min (B), and fragment-ion spectra (MS^2^) for the four major peaks (C). Number 5 refers to supplementary figure 1.

**6**

**B**

**C**

**C**

**C**

**C**

Full scan MS^1^ spectra at 23.6 min (B), and fragment-ion spectra (MS^2^) for the four major peaks (C). Number 6 refers to supplementary figure 1.

**7**

**B**

**C**

**C**

Full scan MS^1^ spectra at 31.7 min (B), and fragment-ion spectra (MS^2^) for the two major peaks (C). Number 7 refers to supplementary figure 1.

**8**

**B**

**C**

**C**

**C**

Full scan MS^1^ spectra at 32.1 min (B), and fragment-ion spectra (MS^2^) for the three major peaks (C). Number 8 refers to supplementary figure 1.

**9**

**B**

**C**

**C**

**C**

Full scan MS^1^ spectra at 32.3 min (B), and fragment-ion spectra (MS^2^) for the three major peaks (C). Number 9 refers to supplementary figure 1.

**10**

**B**

**C**

**C**

Full scan MS^1^ spectra at 45.7 min (B), and fragment-ion spectra (MS^2^) for the two major peaks (C). Number 10 refers to supplementary figure 1.

**11**

**B**

**C**

Full scan MS^1^ spectra at 46.5 min (B), and fragment-ion spectra (MS^2^) for the major peak (C). Number 11 refers to supplementary figure 1.
